# Aucubin Promotes BMSCs Proliferation and Differentiation of Postmenopausal Osteoporosis Patients by Regulating Ferroptosis and BMP2 Signalling

**DOI:** 10.1111/jcmm.70288

**Published:** 2025-01-17

**Authors:** Yang Zheng, Rongtai Sun, Huan Yang, Tianyuan Gu, Meichun Han, Congcong Yu, Pengyu Chen, Jianhua Zhang, Ting Jiang, Yangyang Ding, Long Liang, Renfu Quan, Shasha Yao, Xing Zhao

**Affiliations:** ^1^ Department of Orthopaedic Surgery, Sir Run Run Shaw Hospital Zhejiang University School of Medicine Hangzhou China; ^2^ Research Institute of Orthopedics The Affiliated Jiangnan Hospital of Zhejiang Chinese Medical University Hangzhou China; ^3^ Liangzhu Laboratory Zhejiang University Medical Center Hangzhou China; ^4^ Third Clinical Medical School Zhejiang Chinese Medical University Hangzhou China; ^5^ Department of Orthopedics The First Affiliated Hospital of Anhui University of Traditional Chinese Medicine Hefei China

**Keywords:** aucubin, BMP2, BMSC, ferroptosis, postmenopausal osteoporosis, ROS

## Abstract

Postmenopausal osteoporosis (PMOP) is a chronic systemic bone metabolism disorder. Promotion in the patterns of human bone marrow mesenchymal stem cells (hBMSCs) differentiation towards osteoblasts contributes to alleviating osteoporosis. Aucubin, a natural compound isolated from the well‐known herbal medicine Eucommia, was previously shown to possess various pharmacological effects. However, its effects on hBMSCs of PMOP patients are unknown. The aim of this present research was to investigate the impact and underlying process of aucubin on cell proliferation and osteogenic differentiation in hBMSCs isolated from PMOP patients. The ability of aucubin to inhibit the ferroptosis induced by erastin in hBMSCs was detected; ROS production, ferrous ion levels, SOD, MDA, and GPX activities were tested by using commercial kits. Next, ALP staining, ARS staining, RT‐qPCR, RNA‐sequencing, and Western blot were applied for determining the mRNA and protein expression levels associated with the osteogenesis of hBMSCs. The study also explored the involvement of BMP2/Smads signalling in aucubin promoting the osteogenesis of hBMSCs and evaluated the effects of aucubin intervention on osteoporosis using an ovariectomised rat model. The results indicated that aucubin significantly inhibited ROS generation and oxidative stress induced by erastin and protected against ferroptosis in hBMSCs. Additionally, aucubin facilitated osteogenic differentiation of hBMSCs by activating the BMP2/SMADs pathway and attenuated the progression of osteoporosis in OVX rats, suggesting a potential therapeutic benefit for postmenopausal osteoporosis (PMOP).

## 1 Introduction

More than half of postmenopausal women are affected by postmenopausal osteoporosis (PMOP), the most prevalent form of primary osteoporosis. PMOP is primarily caused by a decline in ovarian function, leading to endogenous oestrogen deficiency and characterised by bone mass loss and bone microstructure damage, with a higher rate of bone degeneration exceeding bone formation [1, 2, 3]. Furthermore, PMOP patients suffering from increased bone fragility are at a significant risk of osteoporotic fractures, loss of independence, and mortality. These circumstances place a considerable financial strain on both their families and society as a whole [4]. The detailed pathogenesis of PMOP is currently unknown, but defects of osteogenic differentiation ability and uncontrolled generation of ROS in hBMSCs are closely associated with PMOP [5, 6, 7].

Pluripotent mesenchymal stromal cells, also known as BMSCs, comprise a diverse group of cells with the capacity for self‐renewal and multi‐directional differentiation, encompassing adipocytes, chondrocytes, osteoblasts, and myocytes [8, 9]. BMSCs play a crucial role in regulating bone formation through specific osteogenic signalling pathways and transcription factors, such as the Wnt/β‐catenin, BMP2/SMADs, and Hedgehog pathways, as well as key transcription factors like Runx2 and Osterix [10, 11]. However, the pathways and factors responsible for osteogenic differentiation in BMSCs in postmenopausal osteoporosis (PMOP) are frequently suppressed, leading to impaired bone formation [12]. For instance, research indicates that heightened reactive oxygen species (ROS) production can notably hinder BMSC osteogenic differentiation by inhibiting the Wnt/β‐catenin pathway [13]. Furthermore, the excessive generation of ROS is also a significant factor in the pathogenesis of PMOP. ROS not only causes direct harm to intracellular macromolecules like DNA, proteins, and lipids, but also worsens the impaired osteogenic function of BMSCs through mechanisms such as apoptosis, senescence, and lipid peroxidation. Studies have demonstrated that ROS activation of matrix metalloproteinase‐9 (MMP‐9) leads to degradation of the extracellular matrix and disrupts citric acid metabolism, thereby influencing bone remodelling processes [14]. Another research indicated that elevated levels of ROS have been linked to oxidative stress resulting from prolonged glucocorticoid administration, leading to heightened damage to BMSCs and an augmented susceptibility to bone loss [15].

Ferroptosis, a form of iron‐dependent cell death distinguished by lipid peroxidation resulting from heightened ROS production, has been demonstrated in recent research to significantly influence the osteogenic differentiation of BMSCs, particularly in relation to the development of PMOP [16]. Specifically, excessive iron levels have been shown to trigger BMSC death and impede their osteogenic differentiation by enhancing ROS production and inducing lipid peroxidation [12]. In the context of PMOP, the occurrence of ferroptosis in BMSCs not only contributes to a reduction in bone mass but also exacerbates the progression of PMOP through intricate signalling pathways and transcription factor networks. For instance, the excessive production of reactive oxygen species (ROS) not only directly causes cellular damage but also triggers inflammatory signalling pathways like the Nrf2/HO‐1 pathway, thereby intensifying pathological reactions [17]. These discoveries offer a fresh perspective on comprehending the pathological mechanisms of PMOP and underscore the significant roles of iron‐mediated cell death and oxidative stress responses in PMOP [18, 19, 20]. Therefore, the promotion of osteogenic differentiation and inhibition of excessive lipid ROS production in hBMSCs may be important for the development of new treatments for postmenopausal osteoporosis.

Aucubin (AU), an iridoid glycoside compound, is considered the main component of *Eucommia ulmoides* (Du‐zhong), which is a well‐known kidney‐tonifying Chinese herbal medicine with a long history of safe use for treating bone fractures and osteoporosis in China [21, 22, 23]. However, the effects of aucubin on hBMSCs of PMOP patients have not been evaluated. In this study, we found that aucubin facilitated hBMSCs proliferation by suppressing the excessive production of lipid reactive oxygen species caused by erastin and stimulated hBMSCs osteogenesis through activating the BMP2/SMADs signalling pathway. Experiments in vivo showed that AU mitigates the development of osteoporosis in ovariectomizsd rats. Our work reveals the gene expression pattern and the main mechanism of aucubin promoting hBMSCs osteogenesis and proliferation in patients with PMOP. Furthermore, this research could potentially offer an alternative option for an effective therapeutic method of PMOP.

## 2 Methods

### 2.1 Ethics Statement

The study involving human participants was reviewed and approved by the Ethics Committee of Hangzhou Xiaoshan Hospital of Traditional Chinese Medicine. The study participants gave their consent to participate in this research by providing written informed agreement.

### 2.2 Cell Culture

Mesenchymal stem cells derived from bone marrow (hBMSCs; *n* = 4) were isolated from femur bone marrow aspirates obtained from postmenopausal osteoporosis patients undergoing lower leg fracture surgery with written consent. Briefly, 3–5 mL bone marrow aspirates were plated in T‐25 flasks supplemented with Mesenchymal Stem Cell Growth Medium (90011, Cyagen Biosciences, Guangzhou, China) and kept in a humid incubator at 37°C in an atmosphere of 5% CO_2_ in air for 24 h before the first medium change. The medium was replaced every three days until the cells reached confluence. HBMSCs were dissociated by TryplE (Gibco, cat. 12563029) at 70%–80% coverage and transferred into new cell culture flasks at a ratio of 1:3. And the third passage of cultured BMSCs was used for subsequent experiments. For hBMSCs identification, flow cytometry analysis was performed on hBMSCs from passage 3. The expression of various antigens was examined using phycoerythrin (PE) or fluorescein isothiocyanate (FITC)‐conjugated antibodies, including mouse anti‐human CD29, CD105, CD73, CD90, CD45, CD34, CD11b, and CD14 (Cyagen Chicago, USA). 10,000 cells were collected for each surface marker measurement using a BD C6 Flow Cytometer and BD Accuri C6 software from BD Biosciences. The regions of positive fluorescence were determined by the corresponding isotype‐matched control antibodies. Positive expression was defined as fluorescence exceeding 99% of the corresponding isotype‐matched control antibodies.

### 2.3 Antibodies and Reagents

Cell culture products in our study were purchased from Cyagen (Chicago, USA), including human bone marrow mesenchymal stem cell basal medium and three‐line differentiation medium. Antibodies were purchased from CST (Cell Signalling Technology, MA, USA), Abcam (Cambridge, MA, USA), Boster (Boster Biological Technology, Wuhan, China) and Absin (Absin Bioscience, Shanghai, China). Aucubin (purity > 99%, Yuanye Bio‐Technology, Shanghai, China) was dissolved in phosphate‐buffered saline (PBS), filter‐sterilised, and stored at −20°C. Erastin (MCE, USA) was dissolved in DMSO and stored at −20°C. CCK‐8 kit and alkaline phosphatase (ALP) activity assay kit were obtained from Meilunbio (Dalian, China). Total Superoxide Dismutase Assay Kit, Lipid Peroxidation MDA Assay Kit, Cellular Glutathione Peroxidase Assay Kit, and ARS Staining Kit for Osteogenesis were purchased from Beyotime (Shanghai, China). FerroOrange was obtained from Dojindo (Tokyo, Japan). Bicinchoninic acid (BCA) protein assay kit was obtained from Sangon Biotech (Shanghai, China). Recombinant human noggin protein was obtained from the Absin Bioscience (Shanghai, China).

### 2.4 Cell Viability Assay

Cell Counting Kit‐8 (Meilunbio, Dalian, China) was performed to detect the effects of aucubin on hBMSCs proliferation, following the instructions provided by the manufacturer. Briefly, hBMSCs (1 × 10^3^ cells/well) were placed in 96‐well plates in the BMSC Growth Medium in a humid environment with 5% CO_2_ at a temperature of 37°C. HBMSCs were subjected to various concentrations of AU and erastin for 24, 48, and 72 h. Cells were rinsed with pre‐chilled phosphate buffered saline (PBS), and then 100 μL of fresh BMSCs growth medium containing 10% CCK‐8 solution was added into each hole and incubated for 2 h at 37°C. The absorbance was ultimately measured at 450 nm with a microplate reader (EnSpire, PerkinElmer).

### 2.5 Multi‐Lineage Differentiation of BMSCs In Vitro

To assess osteogenic differentiation, hBMSCs were cultured with varying doses of aucubin (0, 5, 10, and 20 μM) in the presence of osteogenic inducers (Cyagen, Chicago, USA) to identify the optimum level of aucubin (AU) that stimulated osteogenic differentiation of hBMSCs. In order to determine whether the promotion of AU on hBMSCs osteogenic differentiation may be regulated via the promotion of the BMP2/SMADs pathway, we investigated AU impact after inhibiting the BMP2/SMADs pathway with the BMP antagonist, Noggin (Absin). HBMSCs were divided into four groups consisting of three replicates per group to detect the impact of AU (Aucubin) on osteogenesis: Group A was treated with PBS and OIM (osteogenic induction medium); Group B was exposed to AU (10 μM) and OIM; Group C was cultured with a combination of OIM, AU (10 μM) and noggin (200 ng/mL); Group D was treated with OIM and noggin. For adipogenic differentiation, 5 × 10^4^ cells were placed in each well of 6‐well plates and treated with adipogenic induction medium (Cyagen, Chicago, USA). After 3 weeks of treatment, the cells underwent PBS wash, were then fixed using 4% neutral formaldehyde solution, and finally stained with Oil Red O (according to the manufacturer's protocol) to detect lipids. To induce chondrogenic differentiation, cells were seeded at a density of 2 × 10^4^ cells per well in 12‐well plates containing BMSC complete medium. The cells were allowed to reach 70%–80% confluence before aspirating the medium and replacing it with chondrogenic induction medium from Cyagen (Chicago, USA). After 4 weeks of inducement, the monolayer chondrocytes underwent staining using Alcian Blue in accordance with the instructions provided by the manufacturer.

### 2.6 Live/Dead Assay

The live/dead staining was conducted using the Cytotoxicity Assay Kit from Beyotime Biotechnology (Shanghai, China), following the guidelines provided by the manufacturer. In summary, hBMSCs were plated into 24‐wells at a density of 2 × 10^4^ cells per well and treated with varying amounts of aucubin (5 μM and 10 μM) for 72 h. Subsequently, 2.5 μM erastin was added to incubate for 24 h. Then hBMSCs were rinsed with phosphate‐buffered saline (PBS), and the PBS was replaced with 250 μL of fresh Calcein‐AM/PI viability staining solution in each well and incubated for 30 min at 37°C while protected from light. Following PBS washing, the cells were examined and images were captured using an inverted fluorescence microscope from Carl Zeiss in Germany.

### 2.7 Measurement of Oxidative Stress Levels Induced by Erastin

The ROS level of hBMSCs was detected by the DCFH‐DA method using the Reactive Oxygen Species Assay Kit (Beyotime), hBMSCs were exposed to serum‐free medium containing 5 μM DCFH‐DA for 20 min at 37°C while protected from light, and then the cells were rinsed with serum‐free medium twice. The cells were observed and taken photographed using an inverted fluorescence microscope (Carl Zeiss, Germany). Intracellular Fe^2+^ levels were assessed by employing the FerroOrange probe (2 μM, Dojindo). Following various intervention measures, hBMSCs were rinsed with phosphate‐buffered saline (PBS) and exposed to FerroOrange for a duration of 30 min. Subsequently, fluorescence microscopy (Carl Zeiss, Germany) was utilised to capture images, and semi‐quantitative analysis was conducted using ImageJ software. The protein concentration of hBMSCs was detected to normalise the superoxide dismutase (SOD), lipid peroxidation MDA, and glutathione peroxidase (GPX) by BCA protein assay Kit (Sangon Biotech, Shanghai, China). The concentrations of SOD, MDA, and GPX were tested using the corresponding assay kit (Beyotime Biotechnology, China) following the manufacturer's instructions, respectively. The absorbance was tested at 450 nm (SOD), 532 nm (MDA), and 412 nm (GPX) by the microplate reader (EnSpire, PerkinElmer).

### 2.8 Alizarin Red Staining and Mineralisation Assay

Human bone marrow mesenchymal stem cells (hBMSCs) were cultured in 12‐well dishes and exposed to osteogenic induction medium with varying doses of 0, 5, 10, and 20 μM AU. The cells were kept at 37°C and a 5% CO_2_ level for a duration of 21 days. Next, PBS was used to wash all wells three times, followed by fixing them with fixative (Beyotime) for a duration of 20 min. Subsequently, the wells were stained with the ARS Staining Kit (Beyotime) at 37°C for 20 min to observe the calcium formation. Stained hBMSCs were washed with distilled water and detected by light microscope. For quantitative analysis of the mineralisation, 1 mL of a 10% (w/v) solution of cetylpyridinium chloride (Meilunbio, China) was introduced into each well to elute the deposited calcium for a duration of 30 min. Next, the resulting solution was transferred to the 96‐well plate, and the microplate reader measured the absorbance at a wavelength of 570 nm.

### 2.9 Alkaline Phosphatase (ALP) Staining and ALP Activity Assay

Human bone marrow mesenchymal stem cells (hBMSCs) were cultured in 12‐well dishes and exposed to osteogenic induction medium with varying doses of 0, 5, 10, and 20 μM AU at 37°C and 5% CO_2_. For alkaline phosphatase (ALP) staining, the specimens underwent three washes of PBS, followed by fixation with 4% PFA fixative (Beyotime) for 2 min. Subsequently, the specimens were subjected to staining with BCIP (3‐bromo‐4‐chloro‐5‐indolyl phosphate) and NBT (nitro blue tetrazolium) using the Alkaline Phosphatase Colour Development Kit (Meilunbio, China) for 30 min. The cells were rinsed two times with PBS and processed for microscopy analyses. The level of alkaline phosphatase (ALP) activity was assessed using an ALP activity kit (JianCheng Bioengineering Institute, China) as per the instructions provided by the manufacturer. The results were standardised to the corresponding total protein concentration using the BCA assay (Sangon Biotech, Shanghai, China).

### 2.10 RNA‐Seq Library Establishment and RNA‐Seq

The RNA‐Quick Purification Kit (YISHAN Biotechnology, Shanghai, China) was used to extract the total mRNAs of hBMSCs after osteogenic differentiation from three PMOP patients in each group, following the instructions provided by the manufacturer. The quality of RNA was evaluated using an Agilent 2100 Bioanalyzer and verified through RNase‐free agarose gel electrophoresis. Following the extraction of total RNA, eukaryotic mRNA was enhanced using Oligo(dT) beads, whereas prokaryotic mRNA was enriched through the elimination of rRNA using the Ribo‐ZeroTM Magnetic Kit (Epicentre, Madison, WI, USA). Next, the enhanced mRNA was broken down into small fragments using a fragmentation buffer and converted into cDNA through reverse transcription using random primers. DNA polymerase I, RNase H, dNTP, and buffer were used to synthesise the second‐strand cDNA. Afterwards, the cDNA fragments underwent purification using the QiaQuick PCR extraction kit (Qiagen, Venlo, The Netherlands). They were then subjected to end repair, addition of an A base, and ligation with Illumina sequencing adapters. Agarose gel electrophoresis was used to select the size of the ligation products, which were then PCR amplified and sequenced using Illumina Novaseq6000 (Guangzhou Gene Denovo Biotechnology Co. Ltd., Guangzhou, China).

### 2.11 Animal Experiments

The animal experiments were conducted in accordance with the guidelines outlined by the National Institutes of Health's Guide for the Care and Use of Laboratory Animals and were approved by the Ethics Committee of Zhejiang Chinese Medical University under Approval No. IACUC—2023055‐12. Specific‐pathogen‐free female Sprague–Dawley (SD) rats, aged 8 weeks and weighing 200–250 g, were randomly allocated into three groups: the sham group (*n* = 6) as the control group, the OVX group (*n* = 6), and the OVX + AU treatment group (*n* = 6). The rats were housed in standard laboratory conditions, including a 12‐h light/dark cycle and standard diet, with three rats per cage. Following anaesthesia, rats in the OVX and OVX + AU groups underwent ovariectomy, involving the removal of ovaries and their capsules, to induce oestrogen deficiency and osteoporosis model. In the sham group, only a minimal amount of adipose tissue adjacent to the ovaries was excised. One week post‐surgery, rats in the OVX + AU group received intraperitoneal injections of 30 mg/kg of AU every two days, while rats in the Sham and OVX groups were administered 0.9% saline. Following an 8‐week period, the rats were humanely euthanised, and their femurs and tibias were harvested for subsequent micro‐CT imaging and histological evaluation.

### 2.12 Micro‐CT Evaluation and Histological Staining

The femurs were immersed in 4% paraformaldehyde for 24 h for fixation, followed by three washes with PBS. Subsequently, they underwent micro‐CT scanning using a Bruker SkyScan 1174 system at 50 kV voltage and 800 μA current. Three‐dimensional image reconstruction was carried out with N‐Recon software, and subsequent analysis was performed using CTAN software. DataViewer software was utilised to assess bone volume/trabecular volume (BV/TV), trabecular separation (Tb.Sp), trabecular bone thickness (Tb.Th), and bone mineral density (BMD) data. Following fixation with 4% paraformaldehyde, decalcification with EDTA, and paraffin embedding, the tibiae of each group of rats were sectioned into 5 μm slices for haematoxylin and eosin staining, as well as immunohistochemical staining with osteocalcin (OCN) and runt‐related transcription factor 2 (Runx2) (Servicebio, China).

### 2.13 Quantitative Real‐Time Polymerase Chain Reaction (RT‐qPCR) Analysis

RNA extraction was performed on the osteogenic hBMSCs using the RNA‐Quick Purification Kit (YISHAN Biotechnology, Shanghai, China) following the instructions provided by the manufacturer. Primer sequences are listed in Table 1. The RevertAid First Strand cDNA Synthesis Kit (Thermo Scientific Inc., USA) was used to convert total RNAs into cDNA, followed by qPCR using the PowerUp SYBR Green Mix (Thermo Scientific Inc., USA) on the Applied Biosystems 7500 (Thermo Scientific Inc., USA). The mRNA expression levels were standardised to GAPDH using triplicates and calculated using the comparative Ct method (2^−ΔΔCt^). The information was displayed as the ratio of expression change compared to the control group.

**TABLE 1:** Primer and oligo sequences that were used in the study.

Gene	Gene ID	Primer sequence, 5′ to 3′
GAPDH	2597	Forward: CAAGAGCACAAGAGGAAGAGAG
		Reverse: CTACATGGCAACTGTGAGGAG
SLC7A11	23657	Forward: TTACCAGCTTTTGTACGAGTCT
		Reverse: GTGAGCTTGCAAAAGGTTAAGA
SLC3A2	6520	Forward: GGTTCGGGACATAGAGAATCTGAAG
		Reverse: TGCTGAAGGTCGGAGGAGTTAG
PPARG	5468	Forward: TCCGTGGATCTCTCCGTAATGG
		Reverse: TTCTTGTGAATGGAATGTCTTCGTAATG
FABP4	2167	Forward: AAGGCACACGCTCTTGGAATATC
		Reverse: AGAAGCCATCCTCGGACATCAC
OSX	121340	Forward: ATAGTGGGCAGCTAGAAGGGAGTG
		Reverse: ATTAGGGCAGTCGCAGGAGGAG
BGLAP	632	Forward: CTACCTGTATCAATGGCTGGG
		Reverse: GGATTGAGCTCACACACCT
OPN	6696	Forward: TCACACATGGAAAGCGAGGAGTTG
		Reverse: ACTGTCCTTCCCACGGCTGTC
BMP2	650	Forward: GACGTTGGTCAACTCTGTTAAC
		Reverse: GTCAAGGTACAGCATCGAGATA
ID1	3397	Forward: CTACGACATGAACGGCTGTTA
		Reverse: CAACTGAAGGTCCCTGATGTAG

### 2.14 Western Blot

The RIPA lysis buffer, supplemented with 1% PMSF and 1% phosphorylase inhibitor (Beyotime, China), was used to extract the overall protein content of osteogenic hBMSCs. The concentration of protein in each group was measured using a BCA protein kit (Sangon Biotech, Shanghai, China). Around 20 mg of protein, combined with loading buffer (Beyotime), was separated on 8%–20% sodium dodecyl sulfate polyacrylamide gels (SDS‐PAGE) in each lane, which were subsequently transferred onto a PVDF membrane (Millipore, Billerica, USA) for immunoblotting. After membranes were incubated in blocking buffer (TBST [Sangon Biotech, Shanghai, China]) containing 5% skim milk for 1 h at room temperature, the membrane was incubated with the primary antibodies overnight at 4°C. Following three washes with TBST, the blots were visualised using an enhanced chemiluminescence (ECL) kit from (Absin, China). The grey value of each target protein was measured using Bio‐Rad Image Lab software.

### 2.15 Cellular Immunofluorescence

HBMSCs were inoculated and cultured on a cover glass slide, with osteogenic induction commencing upon reaching a cell fusion rate of 80%. Following 10 days of osteogenic induction, the cells were washed three times with phosphate‐buffered saline (PBS). Subsequently, the cells were fixed in 4% paraformaldehyde (Beyotime, China) at room temperature for 20 min, followed by another wash with PBS. The cells were then permeabilized with 0.4% Triton X‐100 (Thermo Fisher) for approximately 1 h and sealed in bovine serum albumin (BSA) buffer at room temperature for about 1 h. The cells were then subjected to incubation with primary antibodies against Runx2 (HUABIO, China) at 4°C for nearly 12 h, followed by incubation with CY3‐conjugated secondary antibodies in the absence of light for approximately 1 h. Subsequently, the cells were washed with PBS and stained with DAPI. The cover glass was sealed with glycerol, and the cells were observed under a fluorescence microscope (Zeiss, Germany).

### 2.16 Statistical Analysis

Bars in all figures represent means ± SEM. A Student's *t* test was performed for comparison between two groups. One‐way ANOVA followed by a post hoc Tukey's test was performed for comparison among multiple groups with GraphPad Prism 8 (GraphPad, USA). *p*‐Values less than 0.05 were considered significant differences.

## 3 Results

### 3.1 Identification of BMSCs

**FIGURE 1:**
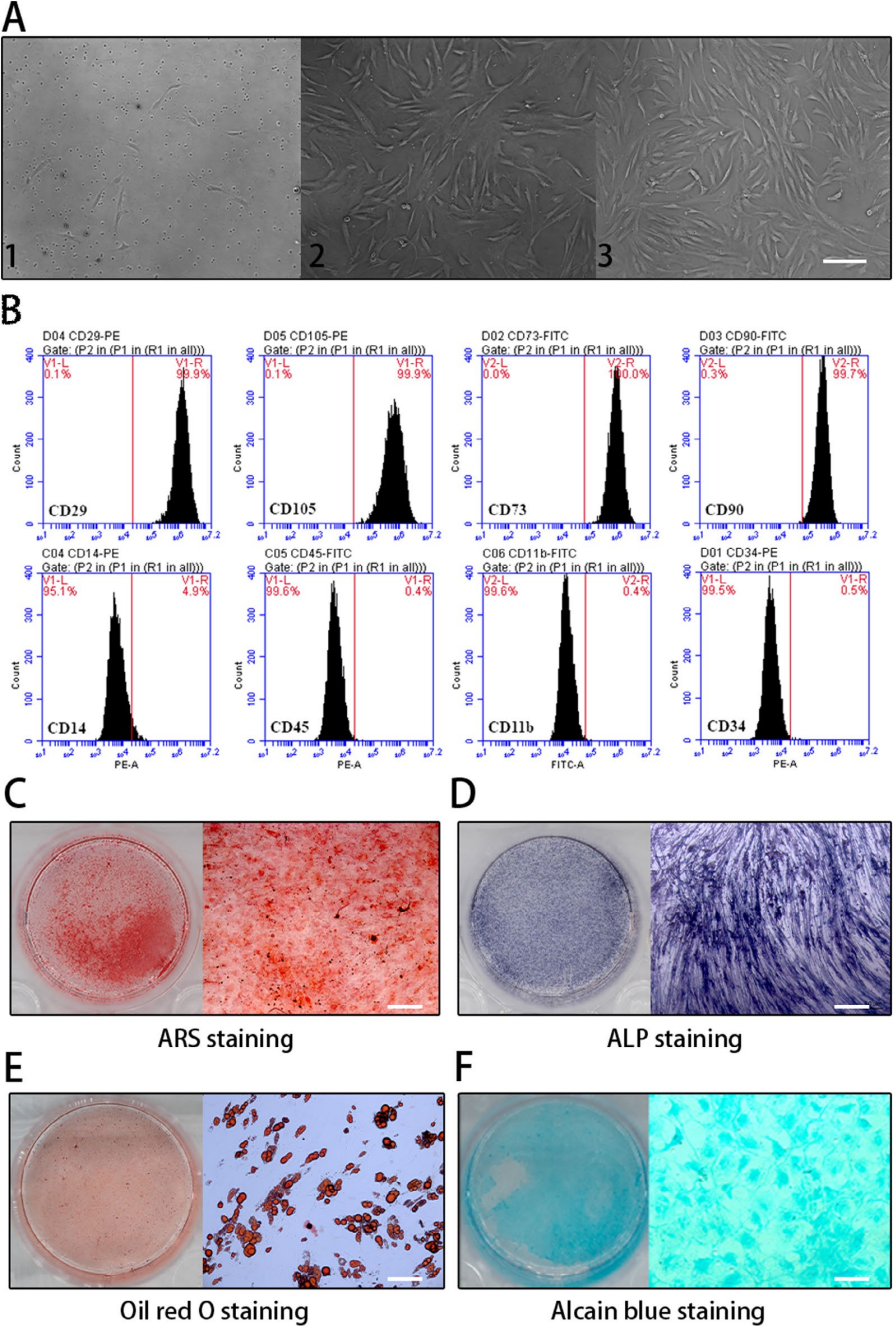
Characterisation of human bone marrow mesenchymal stem cells(hBMSCs). (A) Cell morphology of primary, passage 2, and passage 3 hBMSCs (P0‐5d(A1), P1‐11d(A2), P2‐15d(A3)). Scale bar: 100 μm. (B) Flow cytometry was used to analyse the levels of biomarkers in hBMSCs. The hBMSCs were positive for CD29, CD105, CD90, CD73 and negative for CD34, CD11b, CD45, CD14 companied with corresponding isotype control. (C) Osteoplastic differentiation revealed by alizarin red staining after 3 weeks. Scale bar: 100 μm. (D) Alkaline phosphatase staining after 2 weeks. Scale bar: 100 μm. (E) Adipogenic differentiation revealed by Oil red O staining after 3 weeks. Scale bar: 50 μm. (F) Chondrogenic differentiation revealed by alcian blue staining after 4 weeks. Scale bar: 50 μm.

Following ten days of primary culture, hBMSCs showed a flattened and spindle‐like morphology, which was similar to fibroblastic morphology (Figure 1A), and these cells were expanded and passaged to passage 3 in the standard cell culture media for characterisation. HBMSCs at passage 3 were evaluated by the surface markers CD73(+), CD29(+), CD90(+), CD105(+), CD14(−), CD11b(−), CD34(−) and CD45(−) via flow cytometry. The results demonstrate that the majority of the isolated and purified cells highly expressed typical markers of hBMSCs (Figure 1B). HBMSCs were induced to differentiate into osteoblasts, adipocytes, and chondrocytes with differentiation medium. Osteoplastic cells were detected by alizarin red after 3 weeks (Figure 1C) and alkaline phosphatase (ALP) staining after 14 days (Figure 1D); adipogenic cells were detected by Oil Red O staining after 3 weeks (Figure 1E), and chondrogenic cells were verified by staining of acidic mucopolysaccharides with Alcian Blue after 4 weeks (Figure 1F).

### 3.2 Aucubin Promoted the Proliferation of hBMSCs and Inhibits Erastin‐Induced Viability Reduction

**FIGURE 2:**
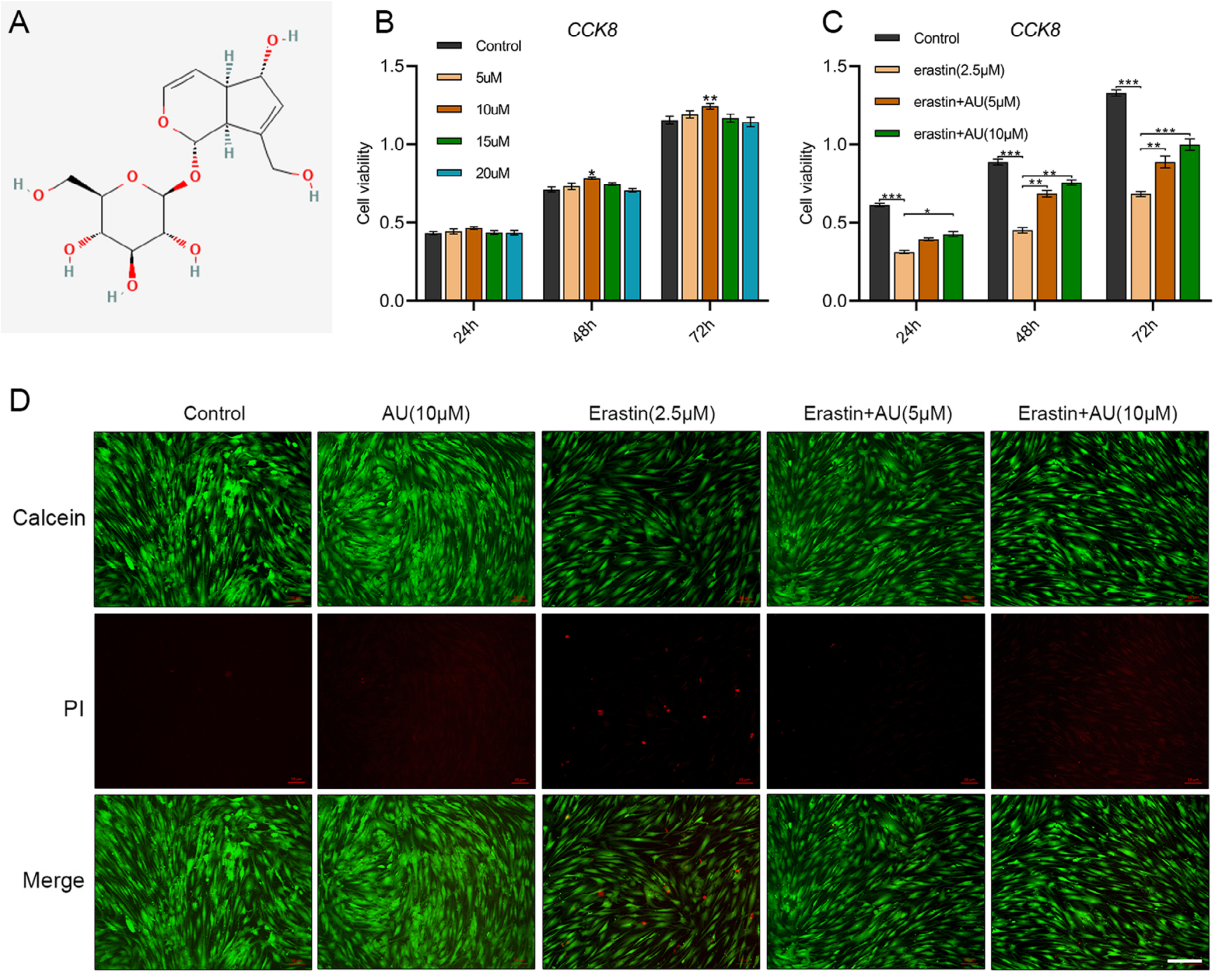
Cell viability of aucubin and erastin on hBMSCs. (A) The chemical structure of aucubin (AU) (PubChem CID:91458). (B) Effect of AU on the proliferation of hBMSCs. hBMSCs were treated with various concentrations of AU (0, 5, 10, 15, and 20 μM) for 24, 48, and 72 h. The proliferation rate of hBMSCs was assessed by the CCK‐8 assay. (C) HBMSCs were treated with or without aucubin (5 and 10 μM) in the presence of erastin (2.5 μM) for 24, 48 and 72 h. (D) Live/dead staining of hBMSCs treated with aucubin (5 and 10 μM) and then coincubating in the presence of erastin (2.5 μM) for 24 h. *N* = 4 in each group. Scale bar: 200 μm.*p < 0.05, **p < 0.01, ***p < 0.001.

To investigate the impact of AU on hBMSCs proliferation, hBMSCs were cultured in basal medium with various doses of AU (0, 5, 10, 15, and 20 μM) and then cell proliferation was measured by the CCK‐8 assay after 24, 48, and 72 h. As shown in Figure 2B, the AU treatment stimulated hBMSCs proliferation at the assigned time points when hBMSCs of PMOP patients were cultured with 10 μM AU. Cytotoxicity Assay Kit (Beyotime) was performed for live/dead staining following the guidelines provided by the manufacturer. Next, hBMSCs were exposed to erastin with or without aucubin for 24, 48, and 72 h (Figure 2C). Moreover, the viability of cells was assessed using live/dead staining after 72 h of treatment (Figure 2D). The staining outcome indicated a notable reduction in the viability of hBMSCs treated with erastin. Moreover, the reduced cell viability was significantly recovered following the addition of aucubin.

### 3.3 Aucubin Alleviated Erastin‐Induced Oxidative Stress of hBMSCs

**FIGURE 3:**
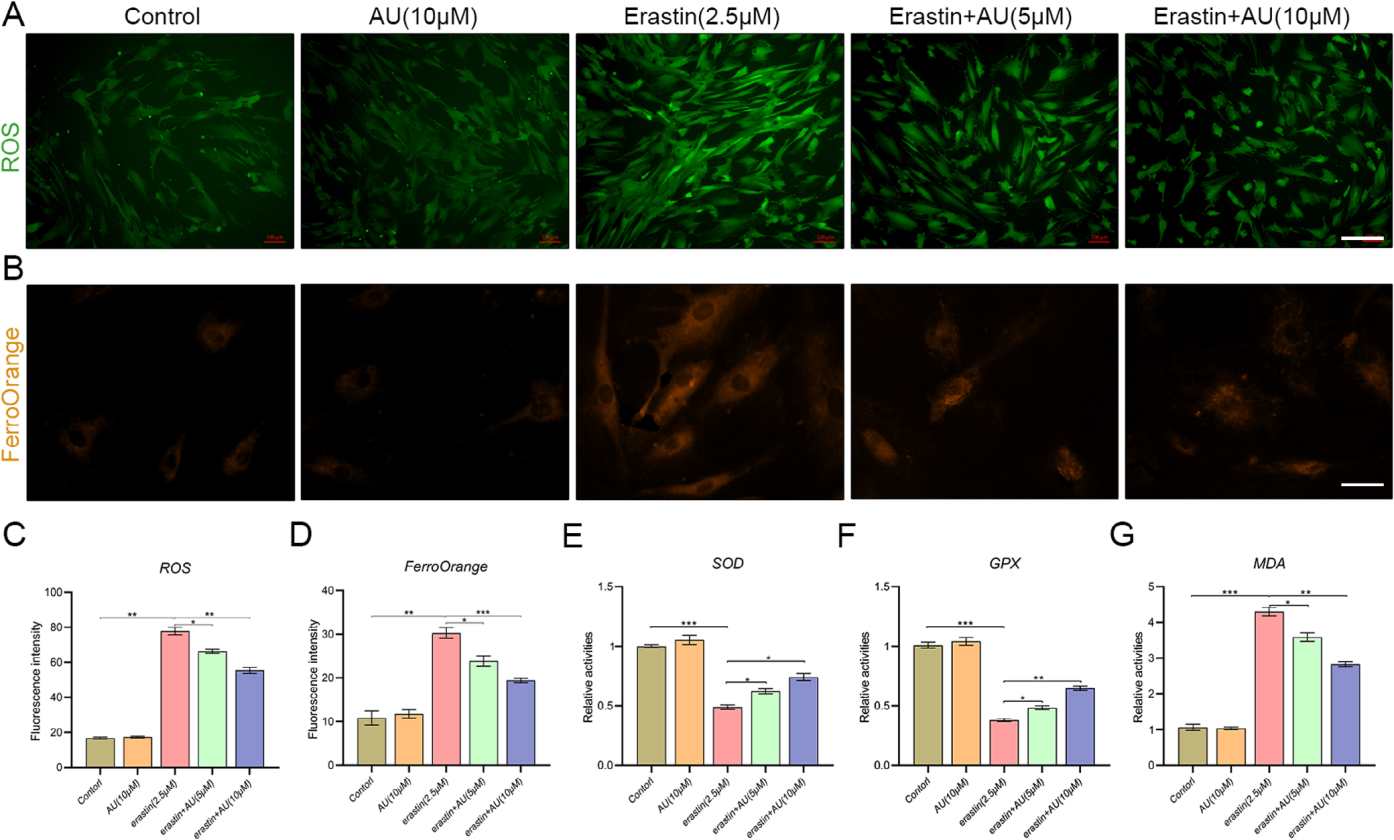
Aucubin alleviates oxidative stress and inhibits lipid accumulation in hBMSCs induced by erastin. (A) Assessing the effect of AU on the production of reactive oxygen species (ROS). Scale bar: 200 μm. (B) Evaluating the influence of AU on intracellular ferrous ion levels in ferroptosis through FerroOrange staining. Scale bar: 50 μm. (C) ROS fluorescence intensity, (D) FerroOrange fluorescence intensity, (E) SOD activity, (F) GPX activity, and (G) MDA levels were measured in hBMSCs (*n* = 4). Scale bar: 200 μm. *p < 0.05, **p < 0.01, ***p < 0.001.

Oxidative stress was detected by measuring the ROS production and the activities of SOD, lipid peroxidation (MDA), and GPX in erastin‐treated hBMSCs. The results revealed that the intracellular ROS levels were significantly increased by treatment with 2.5 μM erastin, while aucubin intervention concentration‐dependently reversed this change (Figure 3A,C). Moreover, exposure to erastin resulted in elevated levels of malondialdehyde (MDA) and intracellular ferrous ions, as well as decreased activity of superoxide dismutase (SOD) and glutathione (GSH) in human bone marrow‐derived mesenchymal stem cells (hBMSCs). Conversely, the presence of aucubin partially mitigated these effects (Figure 3B–G). These findings indicated that the administration of aucubin could suppress erastin‐induced intracellular ROS accumulation and protect hBMSCs from the damage of oxidative stress.

### 3.4 Aucubin Promoted Osteogenic Differentiation of hBMSCs

**FIGURE 4:**
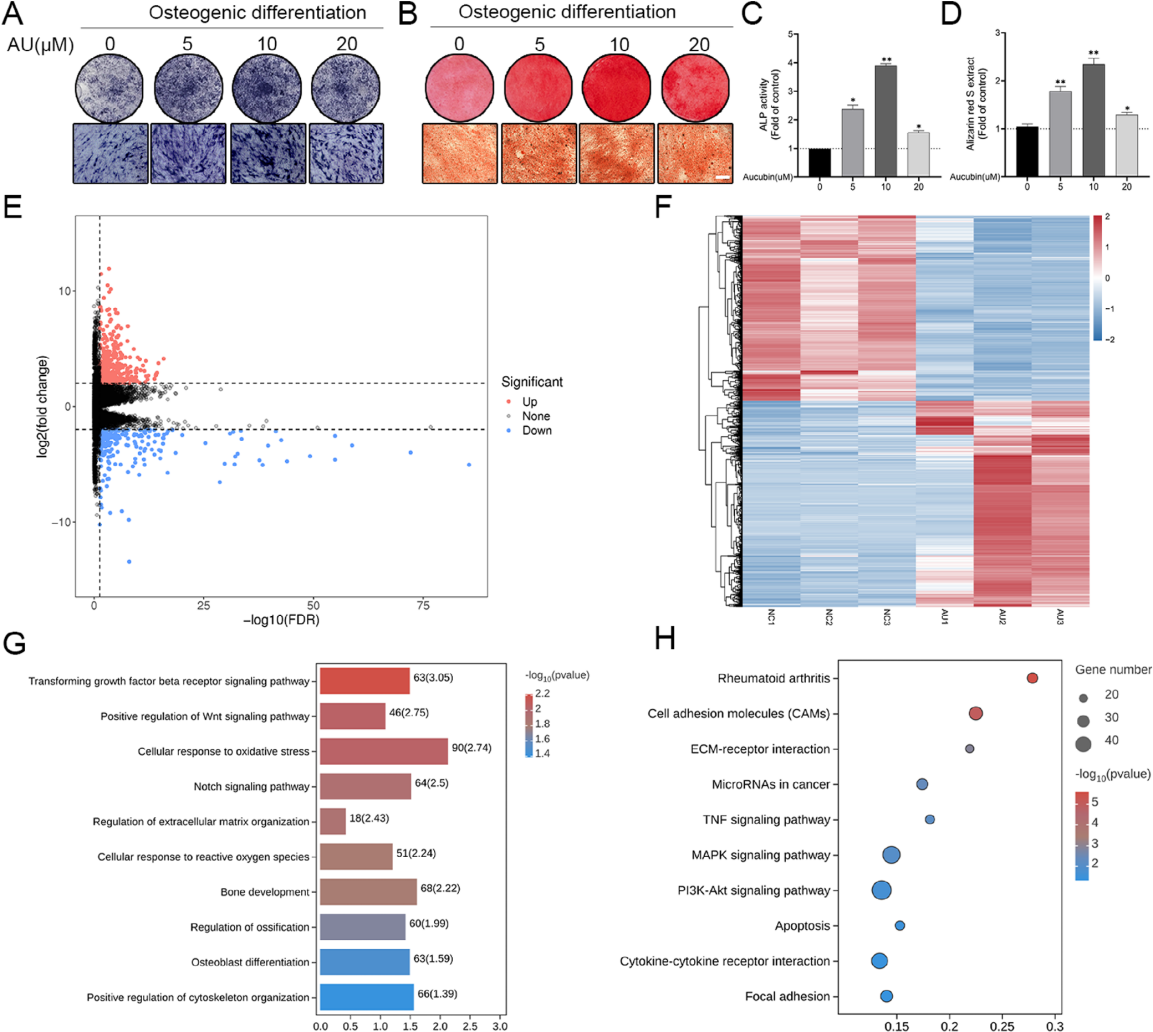
Aucubin enhances the osteoblast differentiation of hBMSCs and RNA‐Seq of aucubin‐treated osteoblasts induced by hBMSCs. (A) The ALP activity of hBMSCs treated with different concentrations of AU was evaluated by ALP staining on 7d. (B) The calcium deposit formation of the four groups were determined by Alizarin red staining on 21d, 10 μM AU treatment significantly increased the osteogenic differentiation of hBMSCs. Scale bar: 200 μm. (C) The ALP activities were quantified using ALP reagent kit. (D) The calcium deposits for matrix mineralization in each group were measured by the cetylpyridinum chloride (CPC) extraction method, with the absorbance of the extracted solution measured at 570 nm. (E) DEGs screened by threshold (adjusted *p* value < 0.05 and |logFC| > 2) were presented by volcano plot. (F) Heatmap displaying the hierarchical clustering of DEGs from NC and AU groups. (G) Bar plot of enriched GO terms involved in biological processes. (H) Bubble plot of enriched KEGG pathway. Larger bubbles indicate a higher number of genes. The colour of each bubble reflects significance. *N* = 3 each group. *p < 0.05, **p < 0.01.

To assess the impact of AU on the osteogenic differentiation of hBMSCs, we conducted Alizarin red staining, ALP staining, and ALP activity assays. HBMSCs were cultured in osteogenic induction medium with or without various doses of aucubin. As shown in Figure 4A,B, aucubin increased the osteogenic activity of hBMSCs for 2 weeks, as evidenced by ALP staining and ALP activity assay, with the most pronounced effect detected for the 10 μM. This outcome was further confirmed by ARS staining and the quantitative analysis of the mineralisation (Figure 4C,D). In short, 10 μM AU significantly promoted the osteogenic differentiation of hBMSCs isolated from PMOP patients. Therefore, 10 μM was selected as the optimal concentration for subsequent experiments.

### 3.5 Examining Gene Expression Profiles of hBMSCs by RNA‐Seq

For investigating the genes that possibly contribute to the promotion of the osteogenic differentiation by AU, we harvested total RNA of hBMSCs after osteogenic differentiation with and without AU (10 μM) for RNA‐seq. Subsequently, we utilised R language to analyse the gene expression profiles. To guarantee the integrity of the data, we apply a filtering process to the initial data prior to conducting bioinformatics analysis to decrease the interference brought by invalid data. To obtain clean reads, we conduct quality control on raw reads and eliminate low‐quality data. The clean reads of each sample of RNA sequencing exceeded 99%, the clean reading Q20% reached 97.0%, and more than 97% of the clean reading data were mapped to the human genome (Table 2). Finally, 19,677 genes were successfully detected from RNA‐Seq. To identify differentially expressed genes (DEGs), we used the criteria of ≥ 2‐fold change and adjusted *p*‐value < 0.05; we subsequently identified a total of 609 DEGs (including 389 up‐ and 220 downregulated), which were further shown in the volcanic map (Figure 4E). A heat map of DEGs between the AU and control groups was then summarised and plotted by hierarchical cluster analysis (Figure 4F).

**TABLE 2:** Quality control information of total reads and mapping ratio in RNA‐Seq.

Sample	Raw data (Mb)	Total clean reads (Mb)	Total clean bases (Gb)	Clean reads Q20 (%)	Total_Mapped (%)
AU1	56.35	56.20 (99.74%)	8.40	97.64	97.55
AU2	42.52	42.41 (99.75%)	6.33	97.58	97.46
AU3	52.27	52.11 (99.69%)	7.80	97.95	97.23
NC1	47.00	46.88 (99.74%)	7.00	97.56	97.36
NC2	62.29	62.08 (99.68%)	9.29	97.88	97.25
NC3	49.91	49.73 (99.63%)	7.44	97.77	97.54

### 3.6 Function and Pathway Analysis of DEGs in AU Group

To further investigate the mechanisms of AU promoting osteogenesis in hBMSCs, GO enrichment analysis of the DEGs was performed in AU and control groups. As shown in Figure 4G, GO analysis showed that the significantly enriched biological processes (BP) of DEGs in the AU and control groups included positive regulation of the Wnt signalling pathway, cellular response to oxidative stress, regulation of ossification, bone development, and the Notch signalling pathway, etc. We continued to apply KEGG pathway enrichment analysis for the DEGs between AU and the control group. The KEGG pathway analysis revealed that DEGs in the AU and control groups were involved in rheumatoid arthritis, Cell adhesion molecules (CAMs), regulation of extracellular matrix organisation, and the MAPK signalling pathway, etc. (Figure 4H).

### 3.7 DEGs Validation of RNA‐Seq Results via RT‐qPCR

**FIGURE 5:**
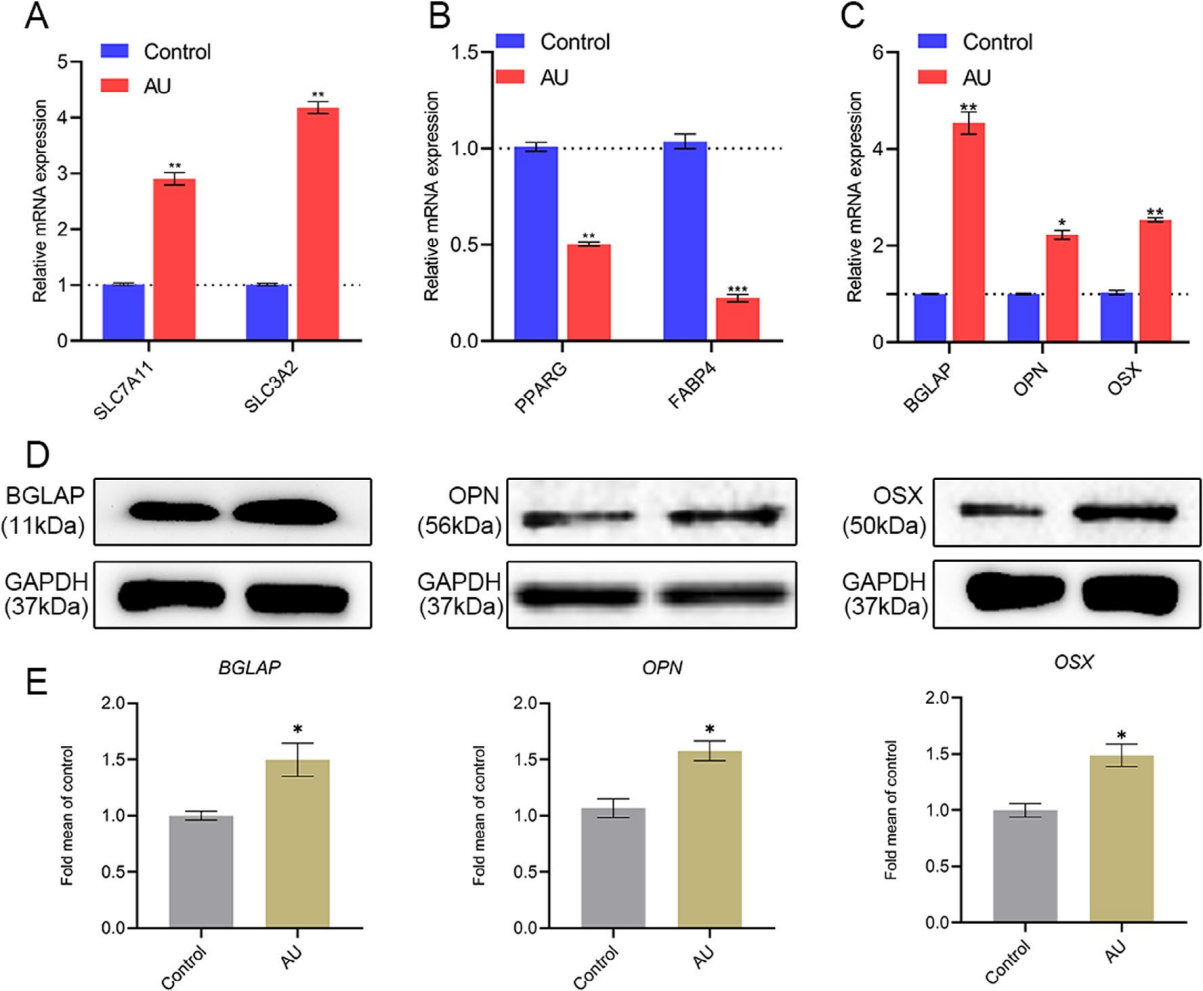
The validation of RNA‐Seq results. (A, B) The expression of two upregulated DEGs (SLC7A11 and SLC3A2) and downregulated DEGs (PPARG and FABP4) from RNA‐Seq was examined by qPCR. (C–E) The expression of some typical genes (BGLAP, OPN, and OSX) involved in osteogenesis was examined by qPCR and western blot. *N* = 3 in each group. *p < 0.05, **p < 0.01, ***p < 0.001 versus the control group. Student's *t*‐test was used for comparison.

Among all DEGs that we detected, some of the DEGs are known to be associated with the osteogenic process, such as BGLAP (bone gamma‐carboxyglutamate protein, fold change = 6.35), OPN (secreted phosphoprotein 1, fold change = 2.40), OSX (Sp7 transcription factor, fold change = 2.09), and BMP2 (bone morphogenetic protein 2, fold change = 2.46). Next, we conducted qPCR to evaluate the reliability of our RNA‐Seq results. 2 upregulated DEGs (SLC7A11 and SLC3A2) inhibiting the ferroptosis and 2 downregulated DEGs (PPARG and FABP4) regulating the adipogenesis with different fold changes were selected to verify their expression by qPCR. QPCR data revealed that the expression of SLC7A11 and SLC3A2 in theAU group was both significantly upregulated (Figure 5A), whereas PPARG and FABP4, which were essential for adipogenesis, were all significantly downregulated in AU‐treated hBMSCs (Figure 5B), which were in accordance with RNA‐Seq results. In addition, we further examined some typical genes participating in osteogenesis by qPCR and western blot, including BGLAP, OPN, and OSX, and the results showed that the expression of BGLAP, OPN, and OSX were all significantly upregulated in the AU group versus the control group (Figure 5C). Western blotting analysis further confirmed the findings at the protein level (Figure 5D,E). Thus, the consequences of qPCR validation indicated that gene expression trends were consistent with those from the RNA‐seq results, indicating that the RNA‐seq data was credible.

### 3.8 Aucubin Promoted Osteogenesis Through BMP2/SMADs Pathway in hBMSCs

**FIGURE 6:**
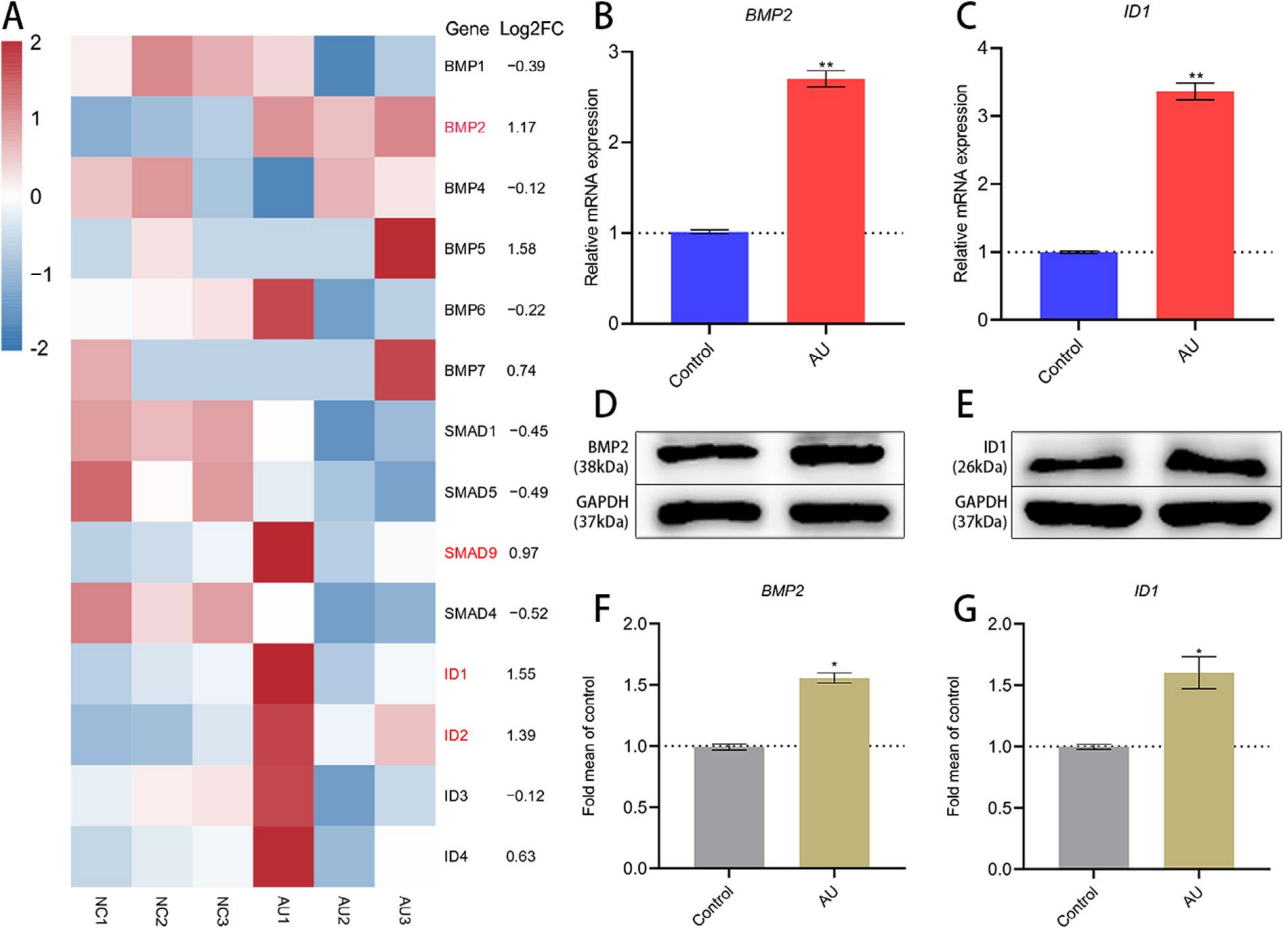
Aucubin treatment stimulated the expression of target genes in the BMP2/SMADs pathway in hBMSCs. (A) Heatmap showing the gene expression of the BMP2/SMADs pathway identified in the aucubin group versus the control group. (B, C) QPCR validation of the upregulation of BMP2 and ID1 genes in the aucubin group versus the control group. (D–G) Protein expression levels of osteogenic factors were measured by Western blotting. *N* = 3 for each group. *p < 0.05, **p < 0.01 versus control group. Student's *t*‐test was used for comparison.

**FIGURE 7:**
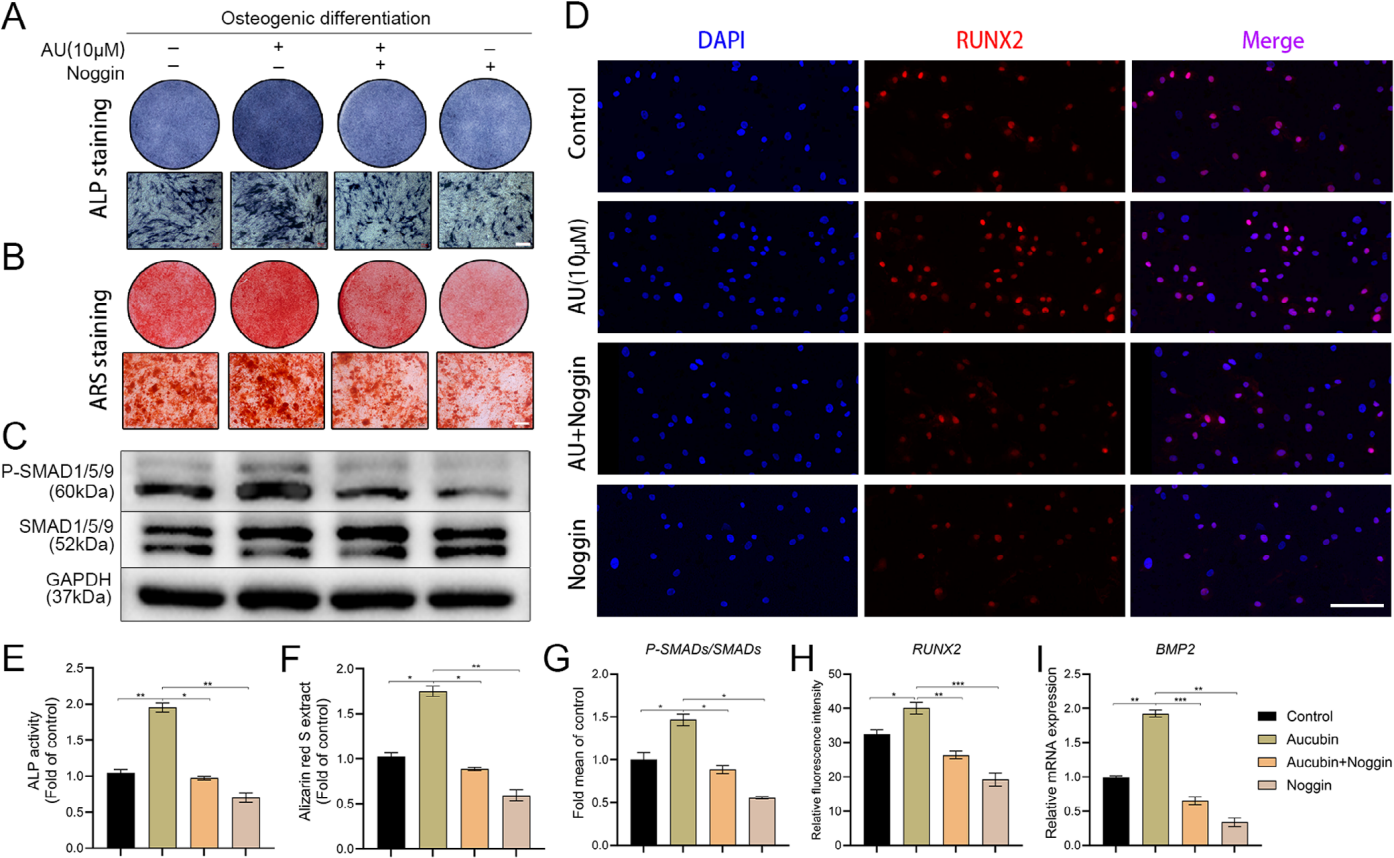
The effects of aucubin on BMP2/SMADs expression in hBMSCs of PMOP patients. (A, E) ALP staining and ALP activity assays were performed on hBMSCs treated with aucubin (10 μM) in the presence or absence of noggin (200 ng/mL). Scale bar: 200 μm. (B, F) Detection of matrix mineralisation by ARS staining and quantification was performed on day 18 of osteogenic differentiation treated with aucubin (10 μM) in the presence or absence of noggin (200 ng/mL). Scale bar: 200 μm. (C, G) Western blot was used to analyse activated (P‐SMAD1/5/9) and total SMAD1/5/9 protein expression of osteoblasts induced by hBMSCs treated with aucubin (10 μM) in the presence or absence of noggin (200 ng/mL), (P‐SMADs/SMADs, the ratio of P‐SMAD1/5/9 to SMAD1/5/9). (D, H) The immunofluorescence analysis was conducted to examine the protein expression of RUNX2 in osteoblasts treated with AU (10 μM) for a duration of 10 days, both in the presence and absence of noggin (200 ng/mL). Scale bar: 100 μm. (I) QPCR was used to analyse the mRNA expression of BMP2 in osteoblasts induced by hBMSCs treated with aucubin (10 μM) in the presence or absence of noggin. *N* = 3 for each group. *p < 0.05, **p < 0.01, ***p < 0.001.

According to the RNA‐Seq results and functional enrichment analysis, we observed BMP2 is significantly upregulated in the AU group (Figure 6A); the BMP2/SMADs pathway plays major roles in osteoblast differentiation and maturation. We therefore further validate the BMP2/SMADs gene expression via qPCR. QPCR results indicated that the mRNA expression of the principal components in BMP2/SMADs is significantly upregulated in the AU group versus the control group of hBMSCs after osteogenic differentiation (Figure 6B,C). Furthermore, western blot validated that the protein expressions of BMP2 and ID1 are remarkably upregulated (Figure 6D–G). Therefore, in order to detect whether BMP2/SMADs signalling mediated AU to promote hBMSCs osteogenesis, we blocked the pathway using noggin protein, an antagonist of the BMP2 /SMADs pathway. ALP and ARS staining indicated that the addition of noggin inhibited the promotion of aucubin on the osteogenic differentiation of hBMSCs (Figure 7A,B,E,F). The results of the western blot confirmed that aucubin induced an obvious increase in the expression of phosphorylated SMAD1/5/9 compared with the untreated control group, while noggin reduced the expression of these proteins (Figure 7C,G). Moreover, the results of immunofluorescence analysis revealed a notable upregulation in the expression of the osteogenic‐specific transcription factor RUNX2 in the group treated with AU compared to the untreated control group, with a contrasting decrease observed in the noggin treatment group. (Figure 7D,H). Additionally, qPCR data indicated a significant downregulation in the mRNA expression of BMP2 in the BMP2/SMADs pathway following noggin treatment (Figure 7I). Collectively, these results revealed that aucubin stimulates osteogenic differentiation in hBMSCs of PMOP patients through the activation of the BMP2/SMADs signalling pathway.

### 3.9 Aucubin Slowed OVX‐Induced Bone Loss in Rat

**FIGURE 8:**
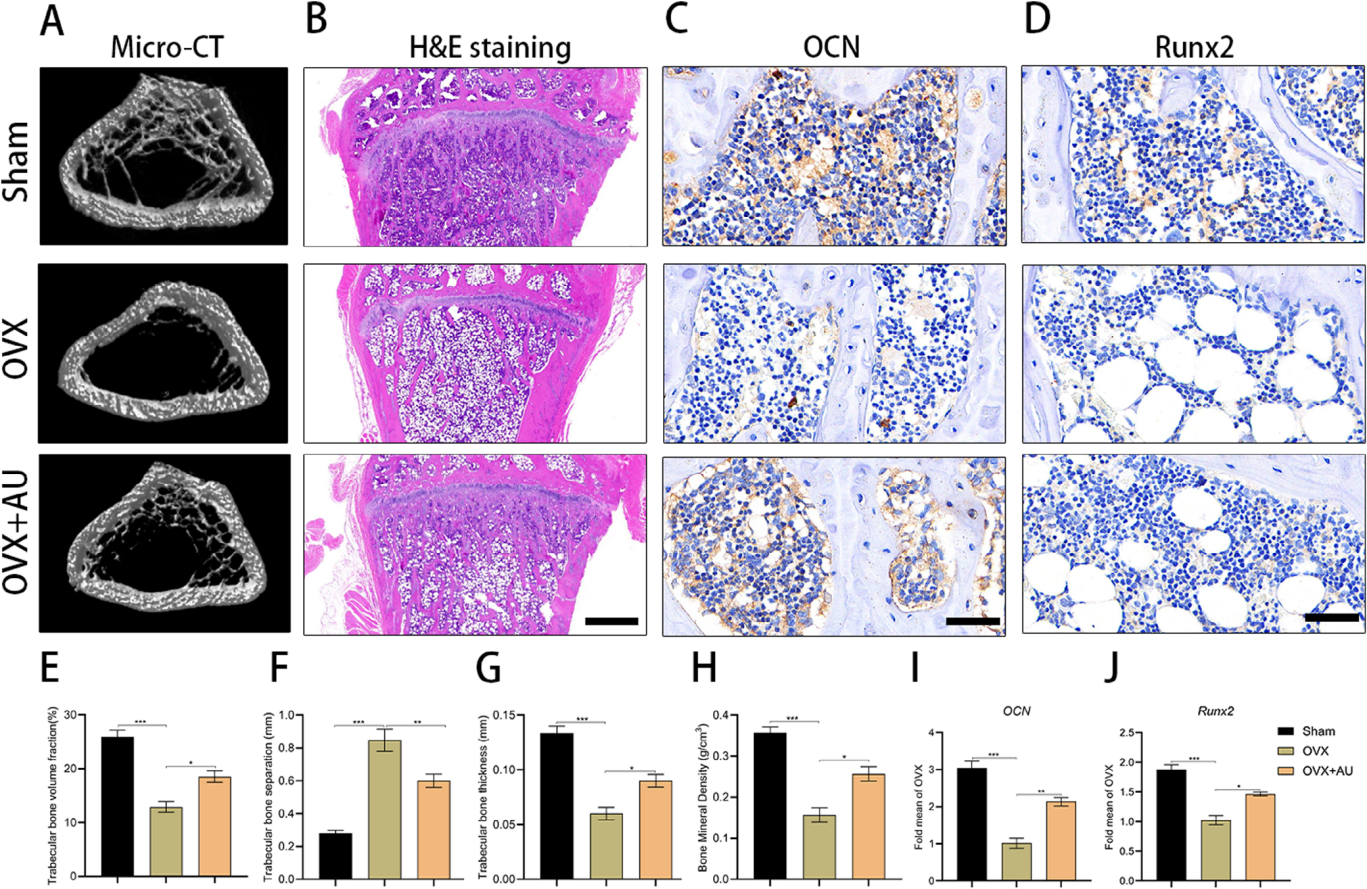
AU alleviates OVX‐induced osteoporosis in rats. (A) Micro‐CT scanning of the distal femurs from rats following intraperitoneal AU injection after the establishment of the osteoporosis model post‐OVX surgery. (B) Coronal sections from the proximal tibia of rats stained with H&E. Scale bar: 1 mm. (C) Osteocalcin (OCN) immunohistochemical staining with brown areas in the coronal sections of the proximal tibia of rats. Scale bar: 50 μm. (D) Runx2 immunohistochemical staining with brown areas in the coronal sections of the proximal tibia of rats. Scale bar: 50 μm. (E–H) Quantitative analysis of the micro‐CT scanning results: BV/TV (E), Tb.Sp (F), Tb.Th (G), and BMD (H). (I) Semiquantitative analysis of OCN staining in IHC. (J) Semiquantitative analysis of Runx2 staining in IHC. *N* = 3 for each group. *p < 0.05, **p < 0.01, ***p < 0.001.

In order to investigate the therapeutic efficacy of AU in managing osteoporosis, intraperitoneal injections of AU (30 mg/kg) were administered to osteoporotic rats every two days for a duration of two months post‐ovariectomy. Subsequent to the treatment regimen, micro‐CT imaging was utilised to evaluate the femoral architecture of the rats. The findings of the study revealed that, in comparison to the OVX group, rats treated with AU exhibited augmented new bone formation in the distal femur, a significant increase in bone volume/trabecular volume (BV/TV), trabecular bone thickness (Tb.Th), and bone mineral density (BMD), as well as reduced trabecular separation (Tb.Sp) (Figure 8A,E–H). H&E staining (Figure 8B) demonstrated higher levels of bone mass and mineralisation in the tibiae of ovariectomised (OVX) rats treated with AU compared to untreated OVX rats. Immunohistochemical (IHC) staining further revealed a significant increase in positive staining areas for osteoblast markers osteocalcin (OCN) and Runx2 at the proximal tibia in the AU treatment group compared to the OVX group (Figure 8C,D,I,J). Overall, these results suggest that intraperitoneal injection of AU effectively promotes osteogenesis and enhances bone mass in OVX rats.

## 4 Discussion

Postmenopausal osteoporosis (PMOP) is a chronic condition affecting the bones, resulting from abnormal bone metabolism and deterioration of bone tissue, contributing to a high risk of fragility fractures and impacting negatively on the quality of life of postmenopausal women [24, 25, 26]. Over the past couple of decades, a great deal of effort has been devoted to the drug treatment of osteoporosis. Currently, pharmacologic agents for osteoporosis are mainly divided into antiresorptive drugs to inhibit bone resorption and anabolic drugs to promote bone formation, including bisphosphonates, denosumab, teriparatide, calcitonin and low‐dose parathyroid hormone [27, 28, 29]. It is widely accepted that postmenopausal osteoporosis is mainly caused by the menopause‐related oestrogen decline, which leads to accelerated bone loss during the reproductive age, and oestrogen replacement therapy acting on osteocytes, osteoclasts, and osteoblasts has gradually shown its therapeutic effect on PMOP, leading to an increase of BMD, inhibition of bone resorption, and maintenance of bone formation [30, 31, 32]. However, their benefits are affected by serious side effects, such as increased risk of coronary, cerebrovascular, and thrombotic events, breast and uterine cancers [33, 34, 35]. Hence, it is crucial to discover medications that have minimal adverse reactions in order to restore the equilibrium between bone resorption and bone formation. Moreover, BMSCs, a cell type frequently employed in tissue engineering, are closely associated with the progression of postmenopausal osteoporosis. The inadequate osteogenic differentiation capability of hBMSCs in PMOP patients compared to those from healthy volunteers [36, 37]. Meanwhile, other studies have identified that ferroptosis could inhibit the proliferation of BMSCs, disturb the equilibrium between bone resorption and bone formation, and further accelerate the occurrence and development of osteoporosis [38, 39, 40].

Recently, an increasing number of researchers have focused on the anti‐osteoporosis effect of traditional Chinese medicine compounds and active components [41, 42, 43]. Aucubin is a major active component that can be extracted from *Eucommia ulmoides* Oliv. (Du‐zhong), which is used in traditional Chinese medicine for treating bone fractures and osteoporosis in China with a long history. Several recent studies have indicated that *Eucommia ulmoides* Oliv. may play a significant role in alleviating osteoporosis development and progression [21, 44]. Aucubin is a natural, stable, and widely used drug. However, to our knowledge, there are few reports on its effect on hBMSCs of postmenopausal osteoporosis patients in the literature. In this study, we found that aucubin could restore hBMSCs viability by antagonising erastin‐induced ferroptosis and enhance osteogenic differentiation by activating the BMP2/SMADs pathway.

Ferroptosis, as a novel form of cell death, is characterised by an iron‐dependent form of programmed and non‐apoptotic cell death that occurs through the lethal accumulation of lipid‐based reactive oxygen species (ROS) when GPX4 or system Xc^−^ activity is inhibited [45]. System Xc^−^ is a cysteine‐glutamate antiporter composed of SLC7A11 and SLC3A2, widely distributed in the phospholipid bilayer, and plays a crucial role in the generation of the antioxidant glutathione [46, 47]. GSH is a tripeptide composed of glutamate, cysteine, and glycine, which are the main antioxidants of protection against oxidative stress‐induced damage [48]. Studies have revealed that inhibition of system Xc^−^ can suppress the exchange of glutathione, leading to the accumulation of intracellular glutamate, decreased intracellular GSH, increased lipid‐based ROS, and ultimately result in ferroptosis [49]. Down‐regulation of SLC7A11 expression suppressed the cystine uptake by the System Xc^−^, thereby decreasing glutathione peroxidase (GPX) activity and cellular antioxidative ability, with increasing ROS‐mediated lipid peroxidation, promoting the occurrence of ferroptosis [50]. Furthermore, one recent study has found YTHDC2 could inhibit SLC3A2 expression by decreasing HOXA13 in an m6A‐indirect manner, inducing lipid peroxidation and ferroptosis [51]. Our current study revealed that adding aucubin in an erastin‐induced cell damage model restored hBMSCs viability, repressed ROS production and intracellular ferrous ions, reduced MDA activity, and promoted SOD and GPX activities. Moreover, RNA‐Seq showed the mRNA expression of SLC7A11 and SLC3A2, which form the System Xc^−^, were both upregulated in the aucubin‐treated hBMSCs, which may be the potential mechanism of antagonising erastin.

Furthermore, we identified that the treatment with aucubin could enhance ALP activity and mineralisation of hBMSCs isolated from PMOP patients. The subsequently examined gene expression, profiles of RNA‐Seq in osteoblasts induced by hBMSCs revealed that aucunbin upregulated the expression of osteogenic genes (BGLAP, OPN) and transcription factor (OSX) while restraining expression of lipogenic gene expression including PAPRG and FABP4. We further explored the molecular interactions among these DEGs by performing functional enrichment analysis and KEGG enrichment analysis, showed that some genes in the BMP2/SMADs pathway were significantly upregulated in the aucubin group. The TGFβ signalling pathway is traditionally categorised into the BMP and activin ligand families, as well as receptors and r‐SMADs. The BMP/Smads pathway is crucial in the regulation of osteoblast differentiation, as well as the development of bone and cartilage [52, 53]. The activation of downstream components of BMP signalling is related to the enhancement of BMP signalling, resulting in the phosphorylation of BMP‐dependent SMAD1/5/9, which complex with SMAD4 and enter the nucleus to induce osteoblast differentiation [54, 55]. Therefore, this pathway is essential for maintaining bone homeostasis under both physiological and pathological conditions. In our study, osteogenic stimulation of aucubin increased the expression of BMP2 and p‐Smad1/5/9, suggesting that aucubin could activate the BMP2 pathway during osteogenesis promotion. In order to examine whether the enhanced osteogenic effect of AU is functioned through the BMP2/SMADs, we blocked the pathway using noggin, an antagonist of BMP2/SMADs signalling pathway. And we found the aucubin‐induced enhancement of osteogenesis was inhibited by noggin, indicating that the ability of aucubin to stimulate osteogenic differentiation in hBMSCs of PMOP patients was mainly dependent on the BMP2/SMADs signalling pathway.

The present investigation has certain limitations. Initially, the study involved a relatively limited number of participants and was conducted specifically with elderly women of Chinese Han nationality. Therefore, more clinical samples need to be collected for further experimental and follow‐up studies to confirm our research. Second, it remains unclear whether there exists a cross‐talk between the BMP2/SMADs pathway and erastin‐induced ferroptosis in the hBMSCs model, which needs further investigation through cell biology research.

In conclusion, the current study demonstrated that aucubin stimulated the proliferation of hBMSCs and enhanced osteogenic differentiation of hBMSCs by protecting against ferroptosis and promoting the activation of BMP2/SMADs signalling. Furthermore, animal experiments conducted in vivo have demonstrated the ability of AU to enhance bone regeneration in ovariectomised (OVX) rats and mitigate the advancement of osteoporosis. These findings suggested that aucubin has anti‐osteoporotic effects in bone marrow mesenchymal stem cells and may be a promising therapeutic agent for postmenopausal osteoporosis patients.

## Author Contributions

**Yang Zheng:** conceptualization (equal), data curation (lead), methodology (equal), project administration (equal), validation (equal), writing – original draft (lead). **Rongtai Sun:** data curation (equal), formal analysis (equal), investigation (equal), methodology (equal), writing – original draft (equal). **Tianyuan Gu:** formal analysis (equal), methodology (equal), visualization (equal), writing – original draft (equal). **Meichun Han:** methodology (equal), validation (equal), visualization (equal). **Pengyu Chen:** formal analysis (supporting), project administration (supporting), validation (supporting), visualization (equal). **Jianhua Zhang:** project administration (supporting), resources (supporting), validation (supporting). **Ting Jiang:** project administration (supporting), resources (supporting), software (supporting), validation (equal). **Yangyang Ding:** resources (supporting), validation (supporting), visualization (supporting). **Long Liang:** investigation (supporting), resources (supporting). **Renfu Quan:** conceptualization (lead), funding acquisition (lead), supervision (supporting), writing – review and editing (lead). **Shasha Yao:** conceptualization (equal), data curation (lead), funding acquisition (equal), resources (equal), supervision (lead). **Xing Zhao:** conceptualization (equal), funding acquisition (equal), investigation (equal), project administration (lead), resources (lead), supervision (equal), writing – review and editing (lead). **Huan Yang:** validation (equal). **Congcong Yu:** validation (equal).

## Conflicts of Interest

The authors declare no conflicts of interest.

## Data Availability

The data that support the findings of this study are available from the corresponding author upon reasonable request.
